# Evaluation of the standard procedure for treatment of periprosthetic joint infections of total knee and hip arthroplasty: a comparison of the 2015 and 2020 census in total joint replacement centres in Germany

**DOI:** 10.1186/s12891-021-04661-3

**Published:** 2021-09-15

**Authors:** Katrin Osmanski-Zenk, Annett Klinder, Christina Rimke, Dieter C. Wirtz, Christoph H. Lohmann, Holger Haas, Bernd Kladny, Wolfram Mittelmeier

**Affiliations:** 1 Orthopädische Klinik und Poliklinik, Universitätsmedizin Rostock, Doberaner Straße 142, 18057 Rostock, Germany; 2 Klinik und Poliklinik für Orthopädie und Unfallchirurgie, Universitätsklinikum Bonn, 53127 Bonn, Germany; 3 Department of Orthopaedic Surgery, Otto-von-Guericke-Universität, 39120 Magdeburg, Germany; 4 Zentrum für Orthopädie, Unfallchirurgie und Sportmedizin, Gemeinschaftskrankenhaus Bonn, 53113 Bonn, Germany; 5 Fachklinik Herzogenaurach, 91074 Herzogenaurach, Germany

**Keywords:** EndoCert, Therapeutic algorithm, Periprosthetic joint infection, Anchoring techniques, International consensus conference

## Abstract

**Background:**

There are different procedures for both, the diagnosis and the therapy of a periprosthetic joint infection (PJI), however, national or international guidelines for a standardised treatment regime are still lacking. The present paper evaluates the use of the predominant treatment protocols for PJI in certified total joint replacement centres (EPZ) in Germany based on an EndoCert questionnaire.

**Materials and methods:**

The questionnaire was developed in cooperation with the EndoCert Certification Commission to survey the treatment protocols for septic revision arthroplasties in EPZ. Questions targeted the various treatment options including prosthesis preserving procedures (DAIR - Debridement, antibiotics, irrigation, and retention of the prosthesis), one-stage revision, two-stage revision, removal of the endoprosthesis and diagnostic sampling prior to re-implantation. All certified EPZ participated (*n* = 504) and the results from the current survey in 2020 were compared to data from a previous analysis in 2015.

**Results:**

The number of centres that performed DAIR up to a maximum of 4 weeks and more than 10 weeks after index surgery decreased since 2015, while the number of centres that provided a one-stage revision as a treatment option increased (hip: + 6.3%; knee: + 6.6%). The majority of the centres (73.2%) indicated a 4–8 week period as the preferred interval between prosthesis removal and re-implantation in two-stage revision in hip as well as knee revisions. Centres with a higher number of revision surgeries (> 200 revisions/year), opted even more often for the 4–8 week period (92.3%). In two-stage revision the use of metal-based spacers with/without reinforcement with antibiotic-containing cement as an interim placeholder was significantly reduced in 2020 compared to 2015. There was also a clear preference for cemented anchoring in two-stage revision arthroplasty in the knee in 2020, whereas the majority of hip replacements was cementless. Additionally, in 2020 the number of samples for microbiological testing during the removal of the infected endoprosthesis increased and 72% of the centres took five or more samples. Overall, the number of EPZ with a standardised protocol for the procedure expanded from 2015 to 2020.

**Conclusion:**

While there was a trend towards standardised therapeutic algorithms for PJI with more uniform choices among the centres in 2020 compared to 2015, the treatment often remains an individual decision. However, since a consistent treatment regime is of vital importance with an expected rise of total numbers of revision arthroplasties, uniform definitions with regard to comparability and standardisation are necessary for the further development of the EndoCert system.

## Summary

This study shows recent changes in therapy algorithms in EPZs with respect to interval duration in two-stage revision, spacer selection, anchoring techniques, diagnostic sampling and duration of antibiotic treatment. In the view of increasing number of revision arthroplasties, therapeutic algorithms and uniform definitions are missing.

## Introduction

Periprosthetic joint infection (PJI) is one of the most serious complications after total joint arthroplasty (TJA) [1, 2], especially when considering the social, economic, psychological and forensic aspects and implications of PJI [3]. Approximately 0.2–2% of patients who undergo primary TJA of the knee or hip joint will develop a PJI [4–6]. The treatment of PJIs poses a complex problem for the orthopaedic surgeon. Not only are there different approaches to diagnosing a PJI [7–11], which in itself is a major challenge, there are also different therapeutic approaches once the diagnosis of PJI is confirmed. These therapies include prosthesis preserving procedures (DAIR - Debridement, antibiotics, irrigation, and retention of the prosthesis) as well as one- and two-stage revisions [2, 6]. Hitherto, there are neither national nor international therapeutic algorithms for the treatment of PJI, although various recommendations were published in recent years [6, 10, 12].

Endoprosthesis centres (EPZ), which were certified under the EndoCert Certification System of the German Society for Orthopaedics and Orthopaedic Surgery (DGOOC), are obliged to provide standardised documentation of their PJI treatment procedure prior to an external audit [13, 14]. These certified centres perform more than 56% of all knee and hip arthroplasties in Germany [15]. Thus, the evaluation of the algorithms in all German certified EPZs offers the possibility to map therapy standards of PJI treatment.

A first evaluation in 2015 already showed differences in therapy standards depending on the size of the centre, i.e. the number of revision cases per centre per year, influenced the choice of procedure [16]. Choices regarding one- or two-stage procedures and the type of anchorage of the final implant were investigated as well as the preferred interval between the two surgeries in two-stage revision.

We hypothesised that the therapeutic algorithms changed between 2015 and 2020, especially with regard to the recommendations of the “Consensus Conference 2018” [10]. The earlier analysis [16] was based on the data of the EndoCert system in the years 2013 and 2014. The aim of the current study was to investigate the development in the therapy of PJI in the certified centres since then by comparing the outcomes of the 2020 questionnaires to the survey from 2015.

## Materials and methods

The Certification Commission of EndoCert developed a standardised questionnaire to assess the treatment for PJI in EPZ [17]. The documentation form on the principles of treatment for PJI has been hardly modified since the start of the pilot project in 2015, so that the results from the survey based on the data years 2013–2014 are comparable with the current analysis (2020) that was based on the data from the years 2018–2019. Every other year the questionnaire has to be completed by the EPZ and must be sent to the independent certification body ClarCert.

The number of cases of multi-site EPZ was summarised, as these centres usually submit just one questionnaire as they follow the same procedure. Only from one multi-site centre, two different questionnaires were available, so that these had to be considered separately in the evaluation.

All centres in Germany that were included in the EndoCert Annual Report 2020 [18] were evaluated. Accordingly, 504 certified EPZs participated in the latest survey while in 2015 a total of 515 centres took part. The data for the evaluation regarding one-stage revision include only facilities that stated in the survey to principally perform one-stage revisions (the number of the centres are *n* = 277 for the hip and *n* = 255 for the knee). Apart from one centre, that indicated that it did not perform any two-stage revisions, all further 503 centres carried out two stage revisions and were included in the evaluation of this procedure. The excluded centre refers such cases directly to the cooperating high volume EPZ.

The questionnaire contains five main categories of the different therapeutic options for PJI. These include DAIR, one-stage revision, two-stage revision, removal of the endoprostheses and diagnostic sampling prior to reimplantation. The questions can be answered by single or multiple responses. Individual responses are also possible.

### Statistical analysis

Descriptive statistics were performed using Microsoft Excel 2016. Quantitative characteristics were described, and absolute and percentage frequencies were given for qualitative parameters. Since all certified EPZ were included, this is a comprehensive survey.

For a differentiated evaluation of the results, subgroups were defined and compared to each other. Defining factors of the subgroup analyses were the distinction between EPZ and EPZmax (high volume centres) as well as the number of revision surgeries per centre per year (centres with < 50 revisions/year (*n* = 268), 50–100 revisions/year (*n* = 90), 101–200 revisions/year (*n* = 66) and > 200 revisions/year (*n* = 26)).

Statistical analysis was carried out with IBM SPSS Statistics 27 (IBM Corp., New York, USA). Comparisons between the surveys of 2015 and 2020 as well as the subgroup analyses for the categorical variables were performed by Fischer’s exact test (two categories) or by Pearson’s chi-squared test (more than two categories). For the latter Bonferroni’s test was used as a post hoc test. All *p*-values resulted from two-sided statistical tests and *p* < 0.05 was considered to be significant.

### Ethics approval

The study was approved by the local institutional ethical committee (A2015–0055).

## Results

### Prosthesis preserving procedures (DAIR)

**Fig. 1:**
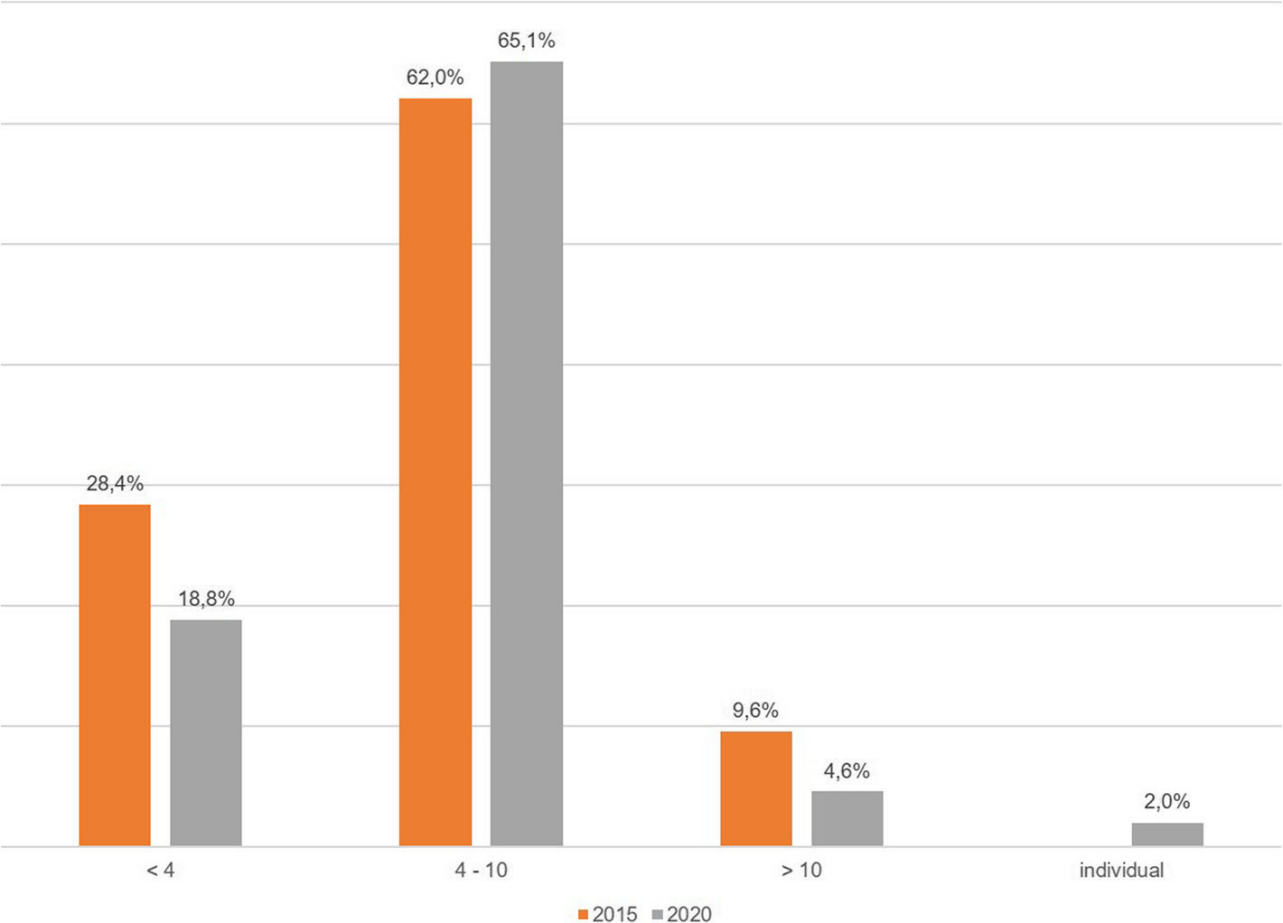
Comparison of the choice of the interval for DAIR

In 2015 and 2020 a similar percentage of centres used DAIR as a treatment option (97.7% (503) and 98.2% (495) for 2015 and 2020, respectively). The majority of centres performed a partial exchange (2015: *n* = 503 (97.7%), 2020: *n* = 490 (97.2%)) as part of DAIR. Furthermore, there were no significant differences between 2015 and 2020 for the preferred time interval after index surgery when the retention of the implant via DAIR is still a viable treatment option in PJI (Pearson’s chi-squared test *p* = 0.168) (Fig. 1).

Currently 65.1% (*n* = 328) of the centres use DAIR treatment in a time frame of 4–10 weeks. The evaluation of 2020 showed that centres with more than 200 revision arthroplasties per year initiate implant-preserving procedure only up to a maximum of 10 weeks after initial implantation, whereas in 2015 still 4.5% of these high volume centres performed the method after more than 10 weeks. At the same time the percentage of these high volume centres that solely used DAIR within the first 4 weeks dropped from 41.0% (*n* = 9) in 2015 to 23.1% (*n* = 6) (Fischer’s exact test *p* = 0.010) in the current evaluation.

### One-stage revision

In the current survey, 255 (50.6%) and 277 (55.0%) centres reported one-stage revisions of total knee and hip arthroplasties, respectively. This was a slight increase compared to 2015 when with 44.0% (*n* = 213) and 48.7% (*n* = 241) less than half of centres carried out one-stage revisions of the knee and hip joint, respectively. While in 2020 these numbers were 6.6% higher for revisions of the knee and 6.3% higher for revisions of the hip joint than 5 years ago, the changes were not significant (Fischer’s exact test *p* = 1.000). Reasons for performing a total one-stage revision are shown in Table 1.

**Table 1:** Reasons for a one-step revision differentiated for joint and number of revisions per year 2020

	EPZ with PJI (*n* = 277)^1^ hip					EPZ with PJI (*n* = 255)^1^ knee				
	< 50	50–100	101–200	> 200	*p*	< 50	50–100	101–200	> 200	*p*
	*n* = 135	*n* = 90	*n* = 38	*n* = 14	Chi^2^	*n* = 125	*n* = 81	*n* = 36	*n* = 13	Chi^2^
Early Infection	80.7%	77.8%	86.8%	78.6%	*0.362*	79.2%	70.4%	80.6%	84.6%	*0.065*
Tissue damage	44.4%^a^	35.6%^a, b^	26.3%^b^	50.0%^a^	***0.003***	42.4%^a, b^	37.0%^a, b^	25.0%^b^	53.8%^a^	***< 0.001***
Age	36.3%^a, b^	26.7%^b^	31.6%^a, b^	50.0%^a^	***0.005***	36.8%^a, b^	28.4%^b^	36.1%^a, b^	53.8%^a^	***0.002***
Causative pathogens	23.7%	28.9%	39.5%	28.6%	*0.104*	24.8%	28.4%	41.7%	30.8%	*0.054*
Others	14.1%	20.0%	18.4%	28.6%	*0.058*	12.0%^a^	21.0%^a, b^	16.7%^a, b^	30.8%^b^	***0.007***

^1^ Number of centres, that performed one-step revision at all. Statistical analysis was performed separately for hip and knee revision surgeries for each of indicated reasons with Pearson’s chi-squared test. Different superscript letters in a row indicate significant differences in the Bonferroni post hoc test between the subgroups

There were no obvious differences regarding the preferred method of implant anchorage in one-stage revision between 2020 and 2015 for surgeries of the hip (2020: cemented *n* = 120 (43.3%) & cementless *n* = 127 (45.8%), 2015: cemented *n* = 115 (47.7%) & cementless *n* = 109 (45.2%); Fischer’s exact test *p* = 0.767) or the knee (2020: cemented *n* = 224 (87.8%) & cementless *n* = 14 (5,5%), 2015: cemented *n* = 196 (92%) & cementless *n* = 8 (3,8%); Fischer’s exact test *p* = 0.534). However, knee implants were significantly more often cemented than hip implants during on-stage revision (Fischer’s exact test *p* < 0.001 for 2015 as well as 2020).

### Two-stage revision

Overall, the duration of the interim interval varied widely between centres. However, there was a wider range in 2020 with a minimal duration of 4 days and a maximum duration of 270 days, while in 2015 the data for the duration between removal and reimplantation ranged from 4 to 120 days. Interestingly for two-stage procedures there was a significant change in the interval between prosthesis removal and reimplantation between 2020 and 2015 (Pearson’s chi-squared test with *p* = 0.003). Bonferroni post hoc testing revealed that the changes were significant for the 4–8 weeks and the 9–11 weeks interval, but not for the interval with more than 12 weeks. While in 2015 only 61.2% (*n* = 315) of the centres favoured the interim interval of 4–8 weeks, this number increased in the current survey to 73.2% (*n* = 368) and 71.8% (*n* = 361) for both hip and knee revisions. Nine to 11 weeks were now preferred by only 2.4% of the centres compared to 16.1% in 2015 and 12 weeks or more were applied only in 3.2% of the centres (2015: 8.3%). When differentiating between EPZ and EPZmax the focus on the 4–8 weeks interval becomes even more apparent (Table 2) and this was also observed in the subgroups regarding the number of revisions, i.e., the more revisions per year were performed in a centre the more likely the centre was to choose an interval of 4–8 weeks. However, the observed differences between EPZ and EPZmax were not significant (Pearson’s chi-squared test with *p* = 0.121 and *p* = 0.202 for hip and knee, respectively).

**Table 2:** Interim interval for two-stage revision differentiated by centre type 2020

	EPZ (*n* = 350)		EPZmax (*n* = 153)	
	hip	knee	hip	knee
< 4 weeks	13.1%	12.0%	4.6%	4.6%
4–8 weeks	69.1%	67.7%	82.4%	81.0%
9–11 weeks	2.6%	2.6%	2.0%	2.0%
≥12 weeks	4.0%	4.0%	1.3%	1.3%
Individual/ individually	7.1%	7.4%	8.5%	8.5%
Not specified	4.0%	6.3%	1.3%	2.6%

Major changes were also evident in the choice of spacer between 2015 and 2020 (Pearson’s chi-squared test with *p* = < 0.001 and *p* = < 0.001 for hip and knee, respectively). In 2015, one-piece metal-based spacers with/without reinforcement with antibiotic-containing cement were used in 40.1% (*n* = 210) of the centres for the hip and in 39.6% (*n* = 204) of the centres for the knee. Five years later, one-piece metal spacers were only used in 8.7% (*n* = 44) of the centres for the hip and in 7.6% (*n* = 38) of the centres for the knee. Usage rates for multi-part metal spacers decreased from 38.1% (*n* = 196) to 8.0% (*n* = 40) of centres for hip revisions and from 39.6% (*n* = 204) to 7.8% (*n* = 29) of the centres for knee revisions between 2015 and 2020. The 2020 survey showed that 63.0% (2015: 61.6%) of the centres used individually shaped cement spacers for the hip and 76.7% (2015: 62.9%) for the knee. Moulded cement spacers are used in 54.7% (2015: 41.7%) of the centres in the hip and 50.3% (2015: 40.8%) in the knee. While in 2015 22.5% of the centres still used permanent resection of the knee prosthesis, this figure was down to 7.2% in 2020. All the detailed changes, that were mentioned above, were significant in Bonferroni post hoc testing.

**Fig. 2:**
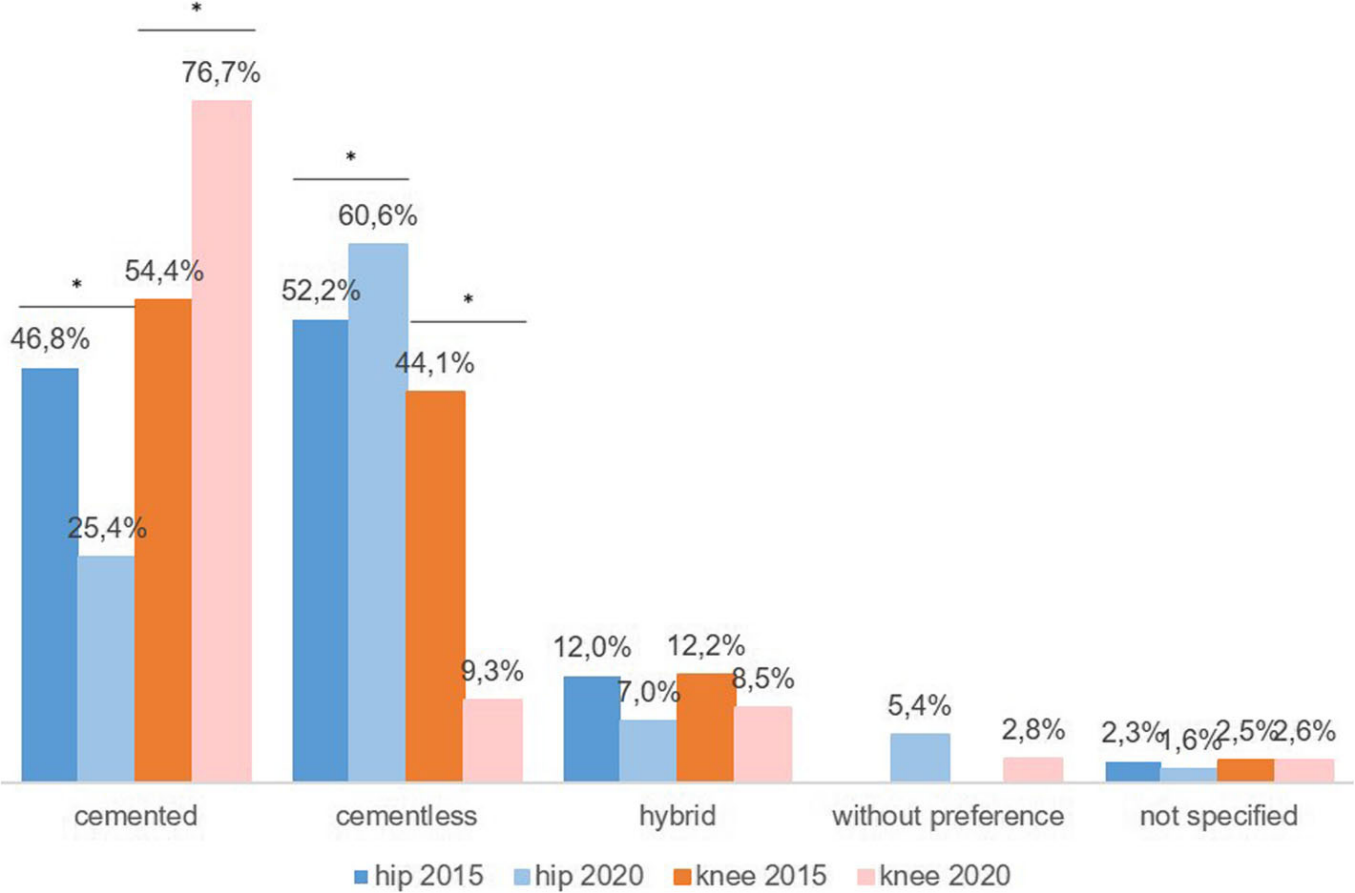
Implant selection for the treatment for the two-stage revision 2015 & 2020

Figure 2 shows the comparison between the different anchoring procedures differentiated by hip and knee as well as the two different survey periods. The comparison of the two periods regarding the preferred cementing type, differentiated by hip and knee shows a significant difference only for the knee (Pearson’s chi-squared test with *p* = 0.064 and *p* < 0.001 for hip and knee, respectively). While 44.1% of centres chose a cementless knee endoprosthesis for reimplantation in 2015, this figure amounted only to 9.3% in 2020. Thus, a clear shift towards a cemented anchorage was detected in the knee.

If the final implant was cemented, premixed cement was used in 75.3% of the centres for hips and in 75.4% for knees in the current survey (2015: 70.7%). Individually mixed cements with specific antibiotic supplementation were used by 16.8% of the centres for the hip and by 17.0% of the centres at the knee (2015: 19.6%). Only a minority of centres used premixed as well as individually mixed cement (2015: 7.6% of the centres; 2020: 5.8% for the hip & 5.2% for the knee).

### Diagnostic sampling in revision surgery and duration of antibiotic therapy

In 2015 37.9% of the centres took 2–4 diagnostic samples intraoperatively, while 58.4% of centres took 5 or more intraoperative samples for microbiological testing during removal of the infected endoprosthesis. In the recent survey only 14.9% of the centres reported taking 2–4 samples whereas 72.0% of centres took 5 or more samples (Fischer’s exact test *p* = 0.001).

In order to test for persistent infection in two-stage revision 315 centres (61.0%) performed a puncture in 2015 whereas in 2020 only 104 centres (20.6%) punctured before the second procedure (Fischer’s exact test *p* < 0.001). In 2015 only 12.4% (*n* = 64) of all centres forwent sampling of any biological material before reimplantation. This figure increased to 32.5% (*n* = 164) in 2020 (Fischer’s exact test *p* < 0.001).

**Fig. 3:**
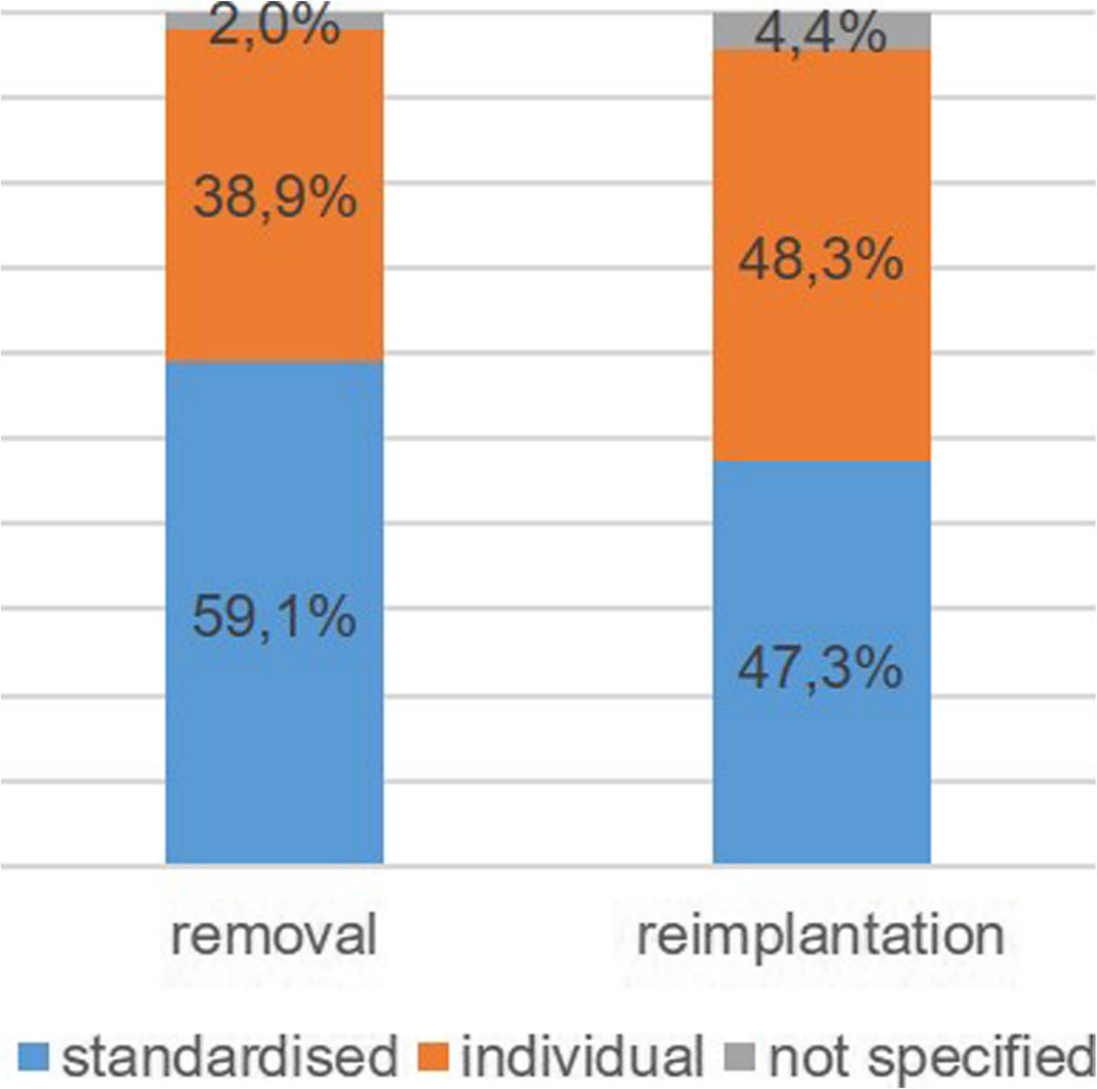
Procedure for the use of antibiotics after removal and reimplantation 2015

**Fig. 4:**
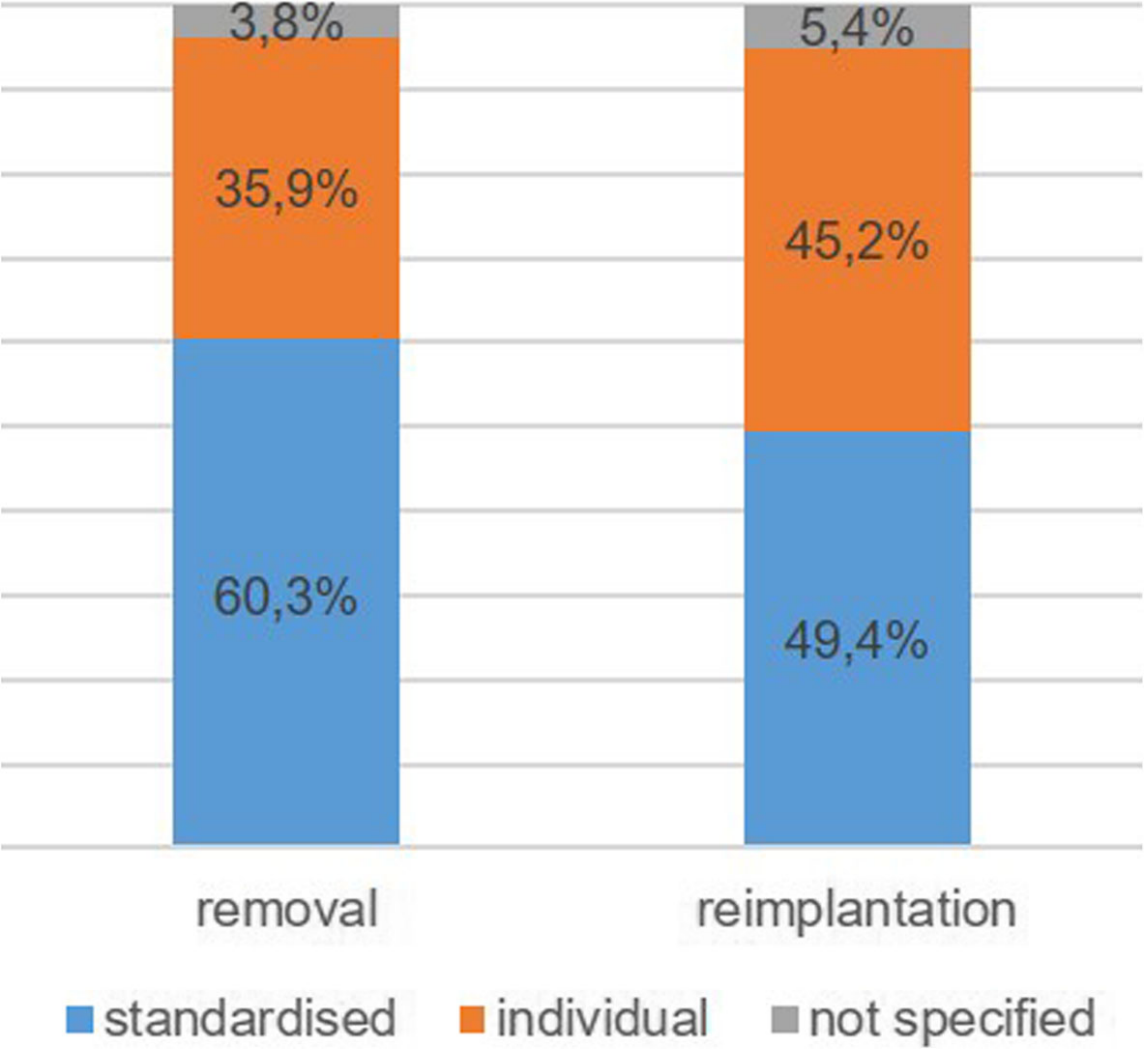
Procedure for the use of antibiotics after removal and reimplantation 2020

The duration of antibiotic administration between removal and reimplantation were stated very differently by the centres in the questionnaires. Some centres named a definite number of days, but many others listed a time span in the questionnaire, so that a direct comparison in days is only possible for a limited number of centres. For this reason, it was additionally considered whether or not a standard procedure was available. The comparison of these data (Figs. 3 and 4) showed no significant changes between 2015 and 2020 (Pearson’s chi-squared test with *p* = 0.672 and *p* = 0.883 for removal and reimplantation, respectively) .

In the evaluation of the 2020 survey it was also determined which of the practised algorithms for antibiotic administration was predominantly used in the centres. The majority of centres (*n* = 210, 21.8%) used postoperative antibiotics for 42 days after removal of the implant and for 42 days after reimplantation of the new endoprosthesis. 3.2% (*n* = 16) of all certified EPZ chose 42 days after removal and 14 days after reimplantation and 7.5% (*n* = 38) of the centres selected 42 days after removal and individual therapy after reimplantation. Twenty seven centres (5.4%) prefered 14 days of antibiotic therapy after removal, but have no standardised timeline after reimplantation.

### Level of standardisation in EPZ

The previous evaluation in 2015 showed that 75% (*n* = 384) of the centres treated PJI according to a standardised algorithm and that the more revision operations per year are carried out in an EPZ the more often a standardised algorithm (> 200 revisions: 87% (*n* = 20)) was applied compared to centres with fewer revisions (< 50 revisions: 72.3% (*n* = 204)) per year. In 2020 86.3% of centres used a standardised approach for at least one of the joints. However, this increase was not significant (Fischer’s exact test *p* = 0.073), probable because the percentage was already high in 2015. For the hip 86.3% (*n* = 434) of the centres had a standardised concept in 2020 and for the knee 84.5% (*n* = 425). Amongst centres with more than 200 revisions per year even 96.2% (*n* = 25) used standardised procedures for both joints, while 83.1% (*n* = 222) and 80.9% (*n* = 216) of centres with less than 50 interventions applied a standardised algorithm for the hip and knee joint, respectively.

## Discussion

The care of patients with PJI requires close cooperation between medical and surgical specialists. Numerous factors, such as duration of the symptoms, age of the implant, causative pathogens, stability of the endoprosthesis, comorbidities of the patient, but also the expertise of the orthopaedic surgeon or the patient’s expectation, influence the decision on the best possible therapy [6].

The definitions of PJI differ significantly and therefore lead to different diagnostic approaches. Nevertheless, in the last decade, various associations and expert groups have provided clear definitions that are internationally recognised. The most important definitions include those of the Musculoskeletal Infection Society (MSIS) [19], the International Consensus Meeting (ICM) [10, 20], the World Association against Infection in Orthopedics and Trauma (WAIOT) [3], the Infectious Diseases Society of America (IDSA) [6] or the European Bone and Joint Infection Society (EBJIS) [21]. Some of those were already revised and updated.

There is still a lack of comprehensive prospective comparative multicentre studies regarding PJI therapy. As a result, an internationally recognised, scientifically proven guideline for the treatment of PJI has yet to be established. Nevertheless, the results of this present study also reflect the efforts to establish standards. The comparison of the two survey periods shows a positive development with regard to establishing a standardised concept in order to deal with septic revisions. Almost all centres that carry out more than 200 revisions per year have established a standardised procedure to treat PJI. This development makes it clear that standardised processes and procedures have become part of everyday hospital life as a consequence of the successful certification by EndoCert.

### Prosthesis preserving procedures (DAIR)

Our current study shows that centres that perform more than 200 revision surgeries per year only applied prosthesis preserving procedures up to a maximum of 10 weeks after index surgery. The predominant time frame is 4–10 weeks while the number of centres that performed prosthesis preserving procedures only up to a maximum of 4 weeks fell from 41 to 23% between 2015 and 2020. However, this trend contradicts the current recommendations from, for example, the 2018 consensus meeting [20] or those of the Trampuz working group [12], who recommend prosthesis preserving procedures up to 30 days after the index surgery or 3 weeks after the onset of symptoms in patients with, among other factors, stable endoprostheses and no sinus tract infection or compromised soft tissue [22]. A limitation of our evaluation regarding the time frame for the use of DAIR is that we did not differentiate between early infection and late acute infection in the questionnaire. Finally, almost all centres (2020: 97.2%) carry out a partial exchange of prosthesis maintenance components. This conforms to the consensus of the Philadelphia Meeting in 2018, where 94% of the delegates were of the opinion that a partial exchange prevents a relapse [23].

### One-stage revision

The comparison of the two evaluation periods shows that over the last 5 years more centres have been using the one-stage revision procedure. This procedure is more common in Europe, while it is rarely applied in the USA [12]. The main reason for this treatment option is early infection. However, the different internationally recognised definitions of early infection can lead to different interpretations and thus therapies [13, 14]. For future evaluations and consequently for a better interpretation of the results, it is therefore recommended to establish a uniform definition of early infection within the certification system.

The type of anchorage used for one-stage replacement did not change over the past years. Implants in the knee joint were mostly cemented while for the hip joint cementless and cemented anchoring techniques were used. During the 2018 consensus meeting 93% of delegates believed that there is no evidence to recommend either cemented or cementless anchorage for the successful treatment of infection. When choosing a cemented endoprosthesis, the incorporation of antibiotics in the cement should also be considered [24, 25]. This recommendation applies to both, the one-stage and the two-stage revision procedure.

### Two-stage revision

The two-stage revision is described in the literature as the gold standard in the treatment of PJI [12, 24]. This is reflected by the results of our current survey. While only 277 centres performed the one-stage revision for the hip joint and 255 centres for the knee joint, almost all centres (*n* = 503) utilised the two-stage revision. This is in agreement with data from the National Joint Registry for England, Wales, Northern Ireland and the Isle of Man between 2003 and 2014 that showed the majority of revision procedures in knee replacements were two-stage revisions with little change between the years [26]. However, a direct comparison of our results with the data of Lenguerrand et al. is not possible, as we recorded the number of centres that performed a certain procedure while Lenguerrand et al. reported the total number of revisions for the procedures DAIR, single-stage, two-stage and others such as arthrodesis in the indicated years.

Li et al. [12] described a short interval of 2–4 weeks between both surgeries (removal and reimplantation) for patients with a known and easy-to-treat bacterial spectrum and no or less compromised soft tissue or sinus tract; or a long interval of 8 weeks for patients with a difficult-to-treat (DTT) bacteria and severely compromised soft tissue. Sukeik et al. [24] recommended an interim interval of 4–6 weeks with antibiotic therapy. Our results show that within the EndoCert system the predominant interim interval in two-stage implant replacement was 4 to 8 weeks (73.2% in 2020, 61.2% in 2015). An interval period of 9 weeks or more were reported to be the median range in two-stage revision surgeries in the UK up to 2014 [26] but seems to have lost in relevance in recent years according to our results. The distinction between EPZ and EPZmax shows this shift even more clearly. More than 80% of all EPZmax prefer an interim interval of 4–8 weeks for a two-stage revision procedure. In the centres with more than 200 revision surgeries per year, more than 90% chose an interval of 4–8 weeks. During the 2018 consensus meeting, delegates were unable to agree on an optimal time interval due to a lack of evidence [27].

The comparison of the two assessed time periods shows that the use of metal-based spacers with/without reinforcement with antibiotic-containing cement as well as permanent resection (Girdlestone procedure) only played a minor role in recent years. Here, there was a clear shift towards either individually shaped cement spacers or moulded cement spacers for both, the hip and knee. In the course of the consensus meeting, no guidance was agreed on this issue and no recommendations were made regarding the use of non-articulating or articulating spacers [28].

Compared to one-stage revisions, two-stage revisions follow a new direction with regard to the concept of anchoring. For the reimplantation of a knee endoprosthesis, cemented anchorage has been preferred by more than three-quarters of all centres in recent years. When changing the hip joint, the trend is moving away from a cemented anchorage towards a cementless one. While the 2018 consensus meeting did not provide recommendations on the type of anchorage due to the lack of randomised studies, there is a trend towards cementless hip joint endoprostheses with good clinical results [25]. This is also reflected by our results. In the case of a cemented procedure, no significant differences have been observed in recent years. A small increase with respect to a ready-mixed cement was observed for both, the hip and the knee. In parallel, the usage rate for individual antibiotic admixture to the cement decreased minimally.

### Removal of the endoprosthesis

Another positive trend was the increase in the number of samples taken for microbiological analysis when removing the endoprosthesis. Almost three quarters of all certified institutions take five or more samples. This development is consistent with the current literature [9, 29].

The information provided by the centres on the duration of antibiosis administered varied considerably. Some centres indicated definite time periods, others minimum or maximum periods, and still others referred directly to, for example, the procedure published by the workgroup of Trampuz that involves extended antibiotic medication after reimplantation [12]. For this reason, the evaluation or comparison of the recording years was only possible to a limited extent. Even the working group of the 2018 consensus meeting could not come up with a clear recommendation regarding the optimal duration of antibiotic administration in the context of the two-stage implant replacement. Nevertheless, the group finally agreed that, based on the current literature, support can be given to a duration of 4–6 weeks after removal [30]. Basically, the results of our study show that established standards for the use of postoperative antibiosis are found unchanged in the EPZ. In the current evaluation, we also investigated which time intervals were applied by the majority of centres in order to identify which of the internationally published procedures and recommendations are preferably implemented nationwide. This shows a clear trend towards the concept published by the Trampuz group (42 days after removal and 42 days before reimplantation) [12]. The high number of individual antibiotic administrations is justified as the therapy depends on the causative pathogen including antibiotic resistances and the recommendation of the microbiologist.

### Sampling before reimplantation

To assess the eradication of the PJI before reimplantation, there is a clear shift away from preservation of biological material or performance of a puncture. The decrease in the number of punctures performed is also consistent with the recommendations by Mühlhofer et al. [9].

To date, various questions regarding the diagnosis and therapy of PJI remain unanswered. Generally, valid therapeutic approaches cannot be applied to all patients and ultimately the care of these patients often remains an individual decision.

## Conclusion

Different definitions of PJI as well as different treatment options for infections pose great challenges for both medical staff and patients when dealing with infections. The developments of the last few years are partially reflected in the procedures for handling PJI in the certified EPZ, but differences can be clearly observed depending on the number of revisions per centre. Even if the trend is towards standardised therapeutic procedures, the treatment often remains an individual decision.

## Data Availability

The consent of EndoCert – an Institution of the German Society of Orthopaedics and Orthopaedic Surgery has been granted. The data was collected and evaluated within the scope of the EndoCert certification. The specially designed questionnaire and the data obtained are stored and available at EndoCert. The data that support the findings of this study are available from EndoCert GmbH but restrictions apply to the availability of these data, which were used under license for the current study, and so are not publicly available. Data are however available from the corresponding author upon reasonable request and with permission of EndoCert GmbH.

## Abbreviations

DAIR: Debridement, antibiotics, irrigation, and retention of the prosthesis
DDT: Difficult-to-treat
EBJIS: European Bone and Joint Infection Society
EPZ: Certified endoprosthesis centres
EPZmax: Certified high volume endoprosthesis centres
ICM: International Consensus Meeting
IDSA: Infectious Diseases Society of America
MSIS: Musculoskeletal Infection Society.
PJI: Periprosthetic joint infection
WAIOT: World Association against Infection in Orthopedics and Trauma
